# Occult clival chordoma initially unrecognized on MRI presenting with cerebrospinal fluid rhinorrhea: a case report

**DOI:** 10.3389/fonc.2026.1870757

**Published:** 2026-07-01

**Authors:** Sufen Huang, Yuzhang Bei, Lifeng Yuan, Ting Su, Shengqiang Zhou

**Affiliations:** 1 Department of Neurology, Liuyang Jili Hospital, Changsha, China; 2 NeuroMedical Center, The Second Affiliated Hospital of University of South China, Hengyang, China; 3 Department of Neurology, Hunan Provincial Hospital of Integrated Traditional Chinese and Western Medicine (The Affiliated Hospital of Hunan Academy of Traditional Chinese Medicine), Changsha, China

**Keywords:** case report, cerebrospinal fluid rhinorrhea, chordoma, purulent meningitis, subtle imaging findings

## Abstract

**Background:**

Cerebrospinal fluid (CSF) rhinorrhea most frequently results from traumatic or iatrogenic skull base disruption. In contrast, tumor-induced spontaneous CSF leaks and the subsequent development of purulent meningitis (PM) are relatively rare and pose significant diagnostic challenges.

Case Presentation: A 39-year-old man presented with acute headache and fever following a two-month history of intermittent, clear unilateral rhinorrhea. Lumbar puncture confirmed PM, and brain magnetic resonance imaging (MRI) revealed left lateral ventricular empyema but no definitive skull-base mass. After achieving clinical stability with a two-week course of targeted antibiotics, the patient underwent endoscopic CSF fistula repair. Intraoperatively, a clival lesion was encountered; histopathological examination (characterized by physaliferous cells and brachyury positivity) confirmed the diagnosis of chordoma.

**Conclusion:**

This case suggests that persistent spontaneous CSF rhinorrhea can be the initial manifestation of an occult skull-base tumor, even in the absence of clear findings on preoperative MRI. In patients with CSF leak complicated by meningitis, management must prioritize infection control while concurrently pursuing a thorough etiologic workup and timely surgical intervention to address both the fistula and any underlying neoplasm.

## Introduction

Chordoma is a rare tumor that originates along the craniospinal axis, accounting for approximately 0.1% to 0.2% of all intracranial tumors (1). It most commonly occurs in male patients between 50 and 60 years of age, while its incidence is extremely low in patients under 40, and typically involves the skull base, sacrum, or spine (1). Persistent cerebrospinal fluid (CSF) leakage is a well-established risk factor for bacterial meningitis, a life-threatening condition that mandates prompt antimicrobial therapy and surgical management (2). While CSF rhinorrhea is typically associated with trauma or prior surgery, spontaneous leaks secondary to underlying tumors are uncommon and may be occult on routine imaging, leading to diagnostic delays (3). Here, we report a case of purulent meningitis (PM) presenting with spontaneous CSF rhinorrhea, in which preoperative imaging showed ventricular empyema without a discernible mass, but a clival chordoma was identified during endoscopic skull base repair.

## Case presentation

A 39-year-old previously healthy male presented to the emergency department with an acute onset of severe headache, fever (38 °C), neck stiffness, and nausea. He reported a two-month history of intermittent, clear right-sided nasal discharge (rhinorrhea), which was exacerbated by coughing or forward bending. Although glucose was detected in the nasal secretions, we acknowledge that this finding alone is not specific for CSF leakage. Because β2-transferrin and β-trace protein assays were not available at our institution, biochemical confirmation could not be obtained. The diagnosis of CSF rhinorrhea was therefore based on the overall clinical presentation, radiological findings, intraoperative confirmation of a skull-base defect with active CSF leakage, and subsequent resolution after surgical repair. Physical examination revealed a Glasgow Coma Scale (GCS) score of 14, signs of meningeal irritation, and persistent unilateral rhinorrhea.

Complete Blood Count revealed significant leukocytosis (white blood cell count: 35.97 × 10^9^/L) with a neutrophilic predominance (neutrophil count: 34.0 × 10^9^/L, comprising 94.4%). Lumbar Puncture yielded CSF with an opening pressure of 14.7 mmHg. The CSF appeared pale yellow and turbid. Analysis showed marked pleocytosis (49,205 × 10^6^/L white blood cells with 81% polymorphonuclear cells), elevated protein (3.38 g/L), profoundly low glucose (0.04 mmol/L), and a chloride level of 117.4 mmol/L (**Table 1**). Neuroimaging with contrast-enhanced brain magnetic resonance imaging (MRI) demonstrated empyema within the left lateral ventricle, consistent with ventriculitis/ventricular empyema. Preoperative skull base MRI identified a T1-weighted imaging (T1WI)-hypointense, T2-weighted imaging (T2WI)-hyperintense lesion within the sphenoid sinus; however, no definitive clival mass or obvious skull base defect was detected (**Figure 1**).

**Table 1:** Cerebrospinal Fluid Findings.

CSF	Exterior condition	Intracranial pressure (7~15 mmHg)	Leucocyte [(0~8) *10^6^/L]	Multinuclear cell (%)	Proteins (0.15 ~ 0.45g/L)	Glucose (2.5-4.5mmol/L)	Chloride (118-128mmol/L)
Admission	Pale yellow, cloudy	14.7	49,205	81	3.38	0.04	117.4
1 week after admission	colourless and transparent	14.7	41	12.2	1.12	2.35	127.6
2 weeks after admission	colourless and transparent	15.44	48	4.2	0.66	2.11	119.9
4 week after admission	colourless and transparent	16.91	37	0	0.53	2.46	124.6
6 week after admission (at discharge)	colourless and transparent	20.59	43	2.3	0.45	2.5	126.6

CSF, Cerebrospinal fluid.

**Figure 1:**
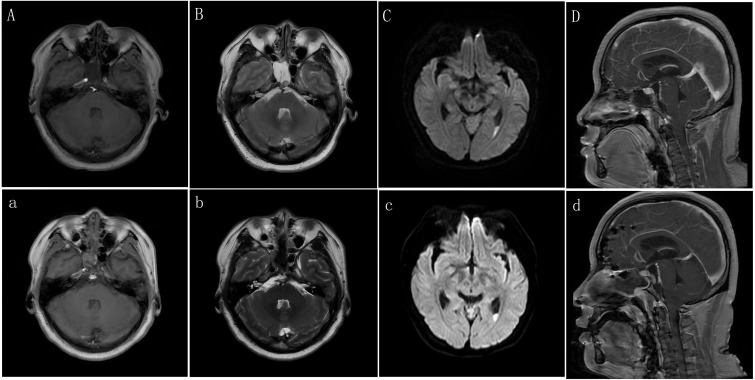
Preoperative skull base MRI: **(A)** Abnormal signal within the sphenoid sinus, appearing heterogeneously hypointense on T1WI. **(B)** Heterogeneously hyperintense signal on T2WI. **(C)** Diffusion-weighted imaging (DWI) reveals hyperintensity in the left lateral ventricle, suggestive of a small amount of ventricular empyema. **(D)** Post-contrast T1WI demonstrates no definite tumor signs and no obvious bony defect in the clivus. Postoperative skull base MRI: **(a)** The previously seen heterogeneously hypointense signal in the sphenoid sinus on T1WI is no longer clearly visualized. **(b)** The previously seen heterogeneously hyperintense signal on T2WI is also no longer clearly visualized. **(c)** DWI still shows a small amount of empyema in the left lateral ventricle. **(d)** Post-contrast T1WI displays postoperative changes in the clivus with no definite tumor visible; mixed high and low signal intensities are noted locally, and multiple air pockets are seen within the surgical site and intracranial cavity.

Empirical intravenous antibiotic therapy with meropenem (2.0g q8h) and vancomycin (1.0g q12h) was initiated. As the lumbar puncture was conducted in the emergency setting, CSF culture was consequently not performed prior to the initiation of antibiotic therapy. Following a two-week course of treatment, the patient showed clinical and CSF improvement, with a follow-up lumbar puncture demonstrating reduced white blood cell count (41×10^6^/L), protein (112.40 mg/dL), and normalized glucose (42.30 mg/dL). Follow-up CSF culture showed no significant abnormalities.

A multidisciplinary plan involving neurosurgery and otolaryngology was developed to address the persistent fistula and investigate its cause. After the infection was controlled, endoscopic fistula repair was performed. During endoscopic endonasal exploration for CSF leak repair, a gelatinous lesion was identified in the sphenoid/clival region, accompanied by underlying clival bone erosion and a dural defect. Multilayer skull base reconstruction was subsequently performed using autologous fascia augmented with a vascularized nasoseptal flap. Intraoperative frozen section analysis of the sampled lesion was conducted, with final pathology confirming the diagnosis of chordoma, characterized by vacuolated (physaliferous) cells within a myxoid stroma and positive nuclear immunoreactivity for brachyury, Epithelial Membrane Antigen(EMA), Cytokeratin(CK), and Vimentin (**Figure 2**). The patient did not receive postoperative radiotherapy.

**Figure 2:**
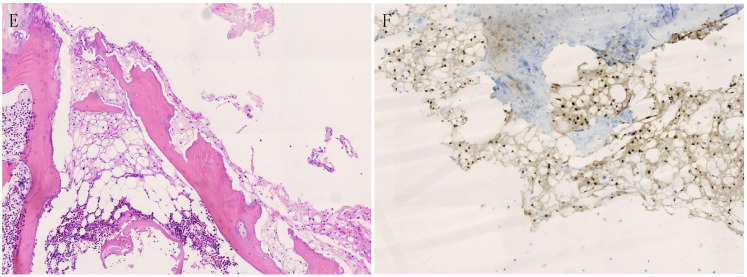
Pathological examination of Clival tissue: **(E)** Haematoxylin and Eosin (HE) staining of the clival lesion reveals a chordoma with small foci of necrosis. The tumor involves cartilage and bone tissue, with formation of small fragments of inflammatory granulation tissue: numerous plasma cells infiltrating the bone marrow cavity (original magnification ×20). **(F)** Immunohistochemical analysis revealed vacuolated (physaliferous) cells within a myxoid stroma, with tumor cell nuclei showing positive immunostaining for brachyury (original magnification ×20).

The patient did not receive postoperative radiotherapy. Postoperative MRI demonstrated gross-total resection without evidence of residual disease. Following multidisciplinary evaluation, close radiological surveillance was selected as the initial management strategy. The patient recovered uneventfully, and follow-up at six months revealed no recurrence of CSF leak, meningitis, or tumor. Given the recognized risk of late recurrence in chordoma, continued long-term oncological surveillance has been maintained.

## Discussion

This case involves a young male who developed PM following two months of persistent unilateral clear rhinorrhea. The initial brain MRI primarily demonstrated pyogenic debris within the left lateral ventricle, and the subtle clival lesion was not appreciated during the initial imaging assessment. After two weeks of antimicrobial therapy, a clival lesion was discovered during endoscopic repair of the CSF rhinorrhea and was subsequently confirmed as a chordoma on histopathological examination. This diagnostic course underscores the challenges associated with recognizing occult clival chordomas presenting with CSF rhinorrhea and meningitis.

Arising from notochordal remnants, clival chordomas are typically characterized by slow and insidious growth along midline neurovascular and osseous structures, often resulting in delayed diagnosis (4). They may remain radiographically occult until causing significant bone erosion or a CSF rhinorrhea, and are sometimes identified incidentally during surgery (5). Although skull base MRI is recommended to rule out clival chordoma in spontaneous CSF leaks, its sensitivity for small lesions remains limited, particularly when masked by inflammation or when using protocols without high-resolution multiplanar sequences (5). Notably, while nuclear brachyury immunopositivity is pathognomonic for chordoma, MRI often fails to detect early-stage tumors, especially in the setting of active infection (6).

Chordomas mediate bone erosion through osteoclast activation and the secretion of matrix metalloproteinases, facilitating the formation of a fistula between the subarachnoid space and the paranasal sinuses (6). Persistent CSF leaks (>7 days) confer a tenfold increased meningitis risk, with Streptococcus pneumoniae being the predominant pathogen in tumor-related cases due to flora migration (7). Given the direct correlation between leak duration and infection, early definitive repair is imperative. Although uncommon, similar presentations have been described previously. Hsieh et al. reported spontaneous CSF rhinorrhea as the initial manifestation of a clival chordoma (8). Alshammari et al. described a patient presenting with CSF rhinorrhea complicated by bacterial meningitis (9). More recently, Tanaka et al. and Prather et al. reported additional cases in which CSF leakage preceded the diagnosis of chordoma (10, 11). These reports collectively demonstrate that CSF rhinorrhea may occasionally represent an early manifestation of clival chordoma and should prompt careful evaluation of the skull base.

The present case shares similarities with these previously reported cases but also illustrates an important diagnostic challenge. Upon retrospective review of the preoperative MRI, a lesion occupying the sphenoid sinus and clivus could be identified as T1 isointense and T2 hyperintense. However, the lesion was subtle and not readily appreciated during the initial evaluation. Unlike the more typical imaging appearance of clival chordoma, which often demonstrates an obvious destructive skull-base mass, the radiological findings in our patient were relatively inconspicuous and could potentially be mistaken for inflammatory or nonspecific sinonasal changes. Consequently, the diagnosis was not established until further investigation was undertaken. This experience highlights the importance of carefully scrutinizing the clival region in patients presenting with unexplained CSF rhinorrhea or recurrent meningitis, even when imaging findings appear subtle.

For cases involving concurrent intracranial infection and suspected or confirmed skull base tumors, infection control must be prioritized before proceeding with definitive resection. Current guidelines recommend a 10- to 14-day course of targeted antibiotic therapy for bacterial meningitis prior to elective skull base surgery, in order to reduce perioperative complication risks (7, 12). The presence of spontaneous CSF rhinorrhea warrants suspicion of an underlying clival chordoma. The preferred treatment for patients with clival chordoma is gross total resection (via endoscopic endonasal surgery when possible) followed by postoperative radiotherapy. Treatment at experienced multidisciplinary cranial base centers is key to minimize complications and to enhance the probability of total removal of the tumors (13). Nevertheless, chordomas carry a poor prognosis owing to their locally aggressive nature and tendency to recur (14), making regular follow-up essential.

Because CSF cultures were not obtained prior to antibiotic administration in the emergency setting, the causative pathogen could not be definitively identified, representing a limitation of the present report. Nevertheless, empirical treatment with meropenem and vancomycin resulted in complete clinical resolution of the infection prior to definitive surgical repair.

Our patient’s preoperative MRI showed only ventricular empyema and no distinct clival mass. Upon retrospective review of the preoperative MRI, a subtle lesion involving the clivus and extending into the sphenoid sinus was identified as T1 isointense and T2 hyperintense. This lesion represented the underlying chordoma but lacked the typical appearance of a destructive clival mass and could easily be mistaken for inflammatory sinonasal pathology. Consequently, it was not recognized during the initial radiological assessment. Possible explanations include small tumor size at imaging, limited skull-base sequences, or tumor obscuration by inflammatory changes. The incidental intraoperative finding altered management: definitive skull-base reconstruction plus oncologic planning for adjuvant therapy.

In retrospect, high-resolution multiplanar CT of the skull base and paranasal sinuses may have provided additional information regarding subtle osseous destruction or skull-base defects that were not readily evident on routine imaging studies. The absence of preoperative high-resolution CT therefore represents a limitation of this report.

This case underscores that spontaneous and persistent CSF rhinorrhea warrants a thorough evaluation of its underlying etiology, even when routine cranial MRI shows only subtle abnormalities in the clival region and lacks definitive or typical imaging features of a skull-base tumor. High-resolution skull-base CT or MRI, endoscopic assessment, and multidisciplinary collaboration may improve the detection of occult lesions. Although rare, clival chordoma should be included in the differential diagnosis of spontaneous CSF rhinorrhea and recurrent meningitis. When secondary bacterial meningitis is present, infection control should be prioritized before definitive repair of the CSF leak. Furthermore, any suspicious lesion encountered intraoperatively should undergo histopathological examination to avoid overlooking an underlying skull-base tumor and to facilitate early diagnosis and appropriate management.

## Conclusion

Persistent spontaneous CSF rhinorrhea may represent the initial clinical presentation of an occult skull base chordoma and can progress to life-threatening PM. When a CSF leak is identified, clinicians should obtain dedicated high-resolution skull base imaging and perform endoscopic evaluation. Active intracranial infection must be controlled prior to surgical repair of the skull base CSF leak. It is regrettable that blood or CSF cultures were not performed before initiating antibiotics in the emergency setting. Such omission should be avoided in future practice. Furthermore, even when preoperative skull-base MRI demonstrates only subtle and non-specific abnormalities, clinicians should maintain a high index of suspicion for rare skull-base tumors, including chordoma, and consider obtaining high-resolution skull-base CT or MRI. Early recognition of this possibility may facilitate appropriate surgical planning and ensure readiness for biopsy or resection if an occult lesion is encountered intraoperatively. Multidisciplinary collaboration is critical to optimize both infection control and long-term oncological management.

## Data Availability

The original contributions presented in the study are included in the article/supplementary material. Further inquiries can be directed to the corresponding author.
